# Spinal TNF-α impedes Fbxo45-dependent Munc13-1 ubiquitination to mediate neuropathic allodynia in rats

**DOI:** 10.1038/s41419-018-0859-4

**Published:** 2018-07-24

**Authors:** Ming-Chun Hsieh, Yu-Cheng Ho, Cheng-Yuan Lai, Dylan Chou, Gin-Den Chen, Tzer-Bin Lin, Hsien-Yu Peng

**Affiliations:** 1 Department of Physiology, College of Medicine, National Taiwan University, Taipei, Taiwan; 2 Department of Medicine, Mackay Medical College, New Taipei, Taiwan; 3 Department of Veterinary Medicine, College of Veterinary Medicine, National Chung-Hsing University, Taichung, Taiwan; 4 Department of Physiology, School of Medicine, College of Medicine, Taipei Medical University, Taipei, Taiwan; 5 Department of Obstetrics and Gynecology, Chung-Shan Medical University Hospital, Chung-Shan Medical University, Taichung, Taiwan; 6 Graduate Institute of Basic Medical Science, China Medical University, Taichung, Taiwan; 7 Department of Bioinformatics and Medical Engineering, Asia University, Taichung, Taiwan; 8 Department of Biotechnology, Asia University, Taichung, Taiwan

## Abstract

Presynaptic active zone proteins play a crucial role in regulating synaptic plasticity. Although the ubiquitin–proteasome system underlying the degradation of the presynaptic active zone protein is well established, the contribution of this machinery to regulating spinal plasticity during neuropathic pain development remains unclear. Here, using male Sprague Dawley rats, we demonstrated along with behavioral allodynia, neuropathic injury induced a marked elevation in the expression levels of an active zone protein Munc13-1 in the homogenate and synaptic plasma membrane of the ipsilateral dorsal horn. Moreover, nerve injury-increased Munc13-1 expression was associated with an increase in the frequency and amplitude of miniature excitatory postsynaptic currents (mEPSCs) in ipsilateral dorsal horn neurons. This neuropathic injury-induced accumulation of Munc13-1 colocalized with synaptophysin but not homer1 in the dorsal horn. Focal knockdown of spinal Munc13-1 expression attenuated behavioral allodynia and the increased frequency, not the amplitude, of mEPSCs in neuropathic rats. Remarkably, neuropathic injury decreased spinal Fbxo45 expression, Fbxo45-Munc13-1 co-precipitation, and Munc13-1 ubiquitination in the ipsilateral dorsal horn. Conversely, focal knockdown of spinal Fbxo45 expression in naive animals resulted in behavioral allodynia in association with similar protein expression and ubiquitination in the dorsal horn as observed with neuropathic injury rats. Furthermore, both neuropathic insults and intrathecal injection of tumor necrosis factor-α (TNF-α) impeded spinal Fbxo45-dependent Munc13-1 ubiquitination, which was reversed by intrathecal TNF-α-neutralizing antibody. Our data revealed that spinal TNF-α impedes Fbxo45-dependent Munc13-1 ubiquitination that accumulates Munc13-1 in the presynaptic area and hence facilitates the synaptic excitability of nociceptive neurotransmission underlying neuropathic pain.

## Introduction

Synaptic vesicle exocytosis is spatially restricted to presynaptic active zone, which is thought to be a central event in neurotransmitter release mediating neuronal communication^1^. Presynaptic active zone proteins are involved in forms of synaptic plasticity by mediating underlying activity-dependent modifications of vesicle release such as organizing vesicle docking and priming^2^. Electrophysiological analysis showed the composition of Munc13 isoforms, a family of active zone proteins, in neurons differentially controls synaptic vesicle priming and subsequently regulates neurotransmission underlying synaptic plasticity^3^. Among the Munc13 family, the role of Munc13-1 in vesicle release-mediated synaptic plasticity has been extensively studied. Electrophysiological studies in murine neuronal cultures and functional analyses in *Caenorhabditis elegans* revealed that Munc13-1 increased the initial synaptic vesicle release probability and synaptic plasticity^4^. The long-term potentiation (LTP)-associated elongation of the presynaptic membrane in the buttons of hippocampal mossy fibers, a morphological synaptic plasticity, was completely blocked in slice cultures of Munc13-1 knockout mutants^5^. Interestingly, the central sensitization, a form of spinal plasticity underlying nociceptive hypersensitivity, relies on molecular processes similar to those involved in LTP development in brain regions;^6^ moreover, one of our recent studies demonstrated that neuropathic pain-associated spinal plasticity involves a presynaptic active zone protein^7^. Nevertheless, the potential involvement of spinal Munc13-1 in the spinal plasticity underlying neuropathic pain development has not been established.

Protein ubiquitination is catalyzed by a cascade of reactions involving a ubiquitin-activating enzyme E1, a ubiquitin-carrier protein E2, and a ubiquitin-protein ligase E3; and the action of each of the various E3s is substrate-specific^8^. Ubiquitin–proteasome systems are currently recognized as having an impact on neurotransmitter release at synaptic sites^9–11^. Recent studies have revealed that F-box and leucine-rich repeat protein 2 (Fbxl2), a synapse-localized E3 ubiquitin ligase, is involved in neural transmission through active zone protein ubiquitination^12^. Our recent study demonstrated that attenuating spinal Fbxl2-mediated ubiquitination of active zone proteins triggered behavioral allodynia in nerve-ligated rats, suggesting that F-box family protein-dependent ubiquitination of presynaptic active zone protein is crucial to neuropathic pain development^7^. Notably, the Drosophila Munc13-1 homolog DUNC13 is degraded by the proteasome, and DUNC13 selectively accumulates in the presynaptic terminal after proteasome inhibition^9–11^. Moreover, a recent study demonstrated that a novel synaptic E3 ligase F-box only protein 45 (Fbxo45) critically regulates neural transmission underlying plastic changes in hippocampal neurons and identified Munc13-1 as a downstream molecule through which Fbxo45 impacts synaptic transmission^13^. Collectively, we hypothesize that Fbxo45 contributes to the spinal presynaptic neurotransmission underlying neuropathic pain development by affecting ubiquitination of Munc13-1.

Tumor necrosis factor-α (TNF-α), a pro-inflammatory cytokine released in response to various neural insults or injury^14^, is proposed to play a key role in neurotransmission^15,16^ and initiation of pain development^17–19^. Our recent results indicated that spinal TNF-α contributes to the development of neuropathic pain through F-box family protein-dependent ubiquitination^20^ and that the F-box family protein modifies presynaptic protein ubiquitination, which is crucial to the development of neuropathic pain^7^. Thus, in the current study, we tested the hypothesis that spinal TNF-α contributes to neuropathic injury-induced behavioral allodynia by modifying Fbxo45-mediated Munc13-1 ubiquitination and degradation.

## Results

### Enhanced Munc13-1 expression in the presynaptic plasma membranes of the dorsal horn following nerve ligation

**Fig. 1:**
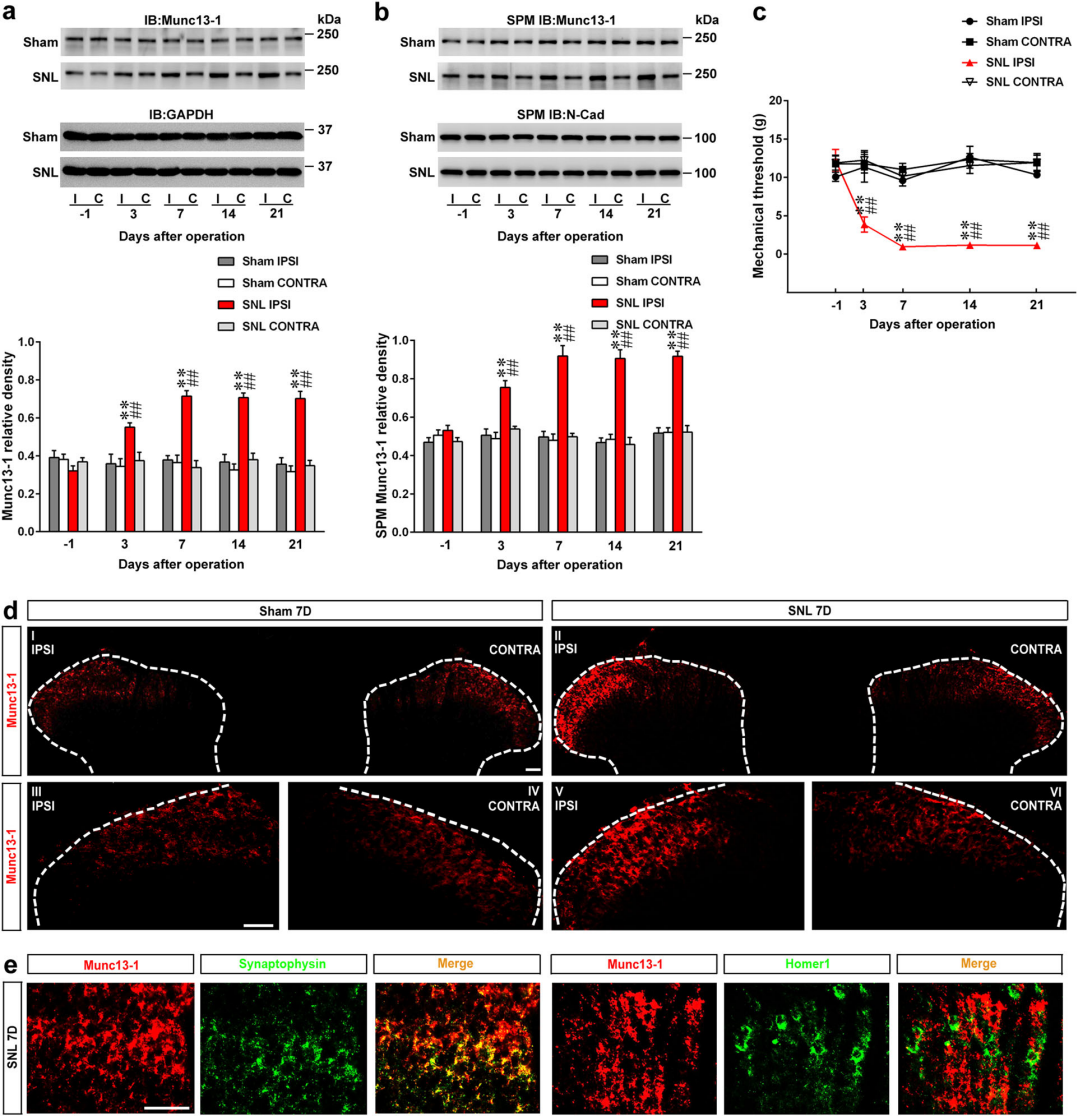
SNL upregulates Munc13-1 expression in presynaptic plasma membranes of dorsal horn accompanied with allodynia. **a**, **b** Representative Western blot and statistical analyses (normalized to GAPDH and N-Cad, respectively) demonstrating that compared with the sham operation group (Sham), spinal nerve ligation (SNL) increased Munc13-1 expression in the total homogenate (**a**) and synaptic plasma membranes (**b**) of the ipsilateral (I and IPSI), but not the contralateral (C and CONTRA), dorsal horn at days 3, 7, 14, and 21 after operation. IB immunoblotting, SPM IB synaptic plasma membranes immunoblotting. **P < 0.01 compared with Sham IPSI. ^##^*P* < 0.01 compared with day −1 (*n* = 6). **c** When compared with the sham operation group, SNL decreased the withdrawal threshold of the ipsilateral (IPSI) but not the contralateral (CONTRA) hind-paw at days 3, 7, 14, and 21 after operation (von Frey test). **P < 0.01 compared with Sham IPSI. ^##^*P* < 0.01 compared with day −1 (*n* = 7). **d**, **e** In the dorsal horn (L4-5) samples. At day 7 post operation, SNL (SNL 7D) increased the immunofluorescence of Munc13-1 (red) in the ipsilateral dorsal horn (IPSI) that colocalized with synaptophysin (green, a presynaptic marker) but not homer1 (green, a postsynaptic marker). Scale bar = 50 μm. Thickness = 30 μm. Results were expressed as mean ± SEM

As a first step in testing the role of spinal Munc13-1 in neuropathic pain development, we conducted Western blot analysis to examine Munc13-1 expression in the dorsal horn homogenate of rats in response to spinal nerve ligation (SNL). SNL significantly increased the amount of Munc13-1 in the ipsilateral, but not the contralateral, dorsal horn on days 3, 7, 14, and 21 after operation (Fig. 1a). Because Munc13-1 expression is spatially restricted to the active zones of the synaptic plasma membrane (SPM)^21^, we next examined Munc13-1 expression in the SPM of the dorsal horn samples using subcellular fractionation analysis. Consistent with the above result observed in the total homogenate, SNL significantly increased the amount of Munc13-1 in the SPM of the ipsilateral dorsal horn on days 3, 7, 14, and 21 after operation (Fig. 1b). The results of the von Frey test revealed that SNL induces behavioral allodynia, as evidenced by a significant decrease in the paw withdrawal threshold on days 3, 7, 14, and 21 after operation (Fig. 1c), which was temporally aligned with enhanced Munc13-1 expression in the dorsal horn. Analogously, images from immunohistochemical analysis revealed that SNL, but not the sham operation, markedly increased Munc13-1-positive immunoreactivity in the ipsilateral dorsal horn on day 7 after operation, while it exhibited no effect on the contralateral side (Fig. 1d). Moreover, double-labeled immunofluorescence staining demonstrated that spinal Munc13-1 colocalized with synaptophysin (a presynaptic marker) on day 7 after SNL but not with Homer1 (a postsynaptic marker; Fig. 1e). Collectively, our data suggest that neuropathic injury-induced nociceptive hypersensitivity is correlated with Munc13-1 upregulation in the presynaptic plasma membranes of the ipsilateral dorsal horn.

### Knockdown of spinal Munc13-1 expression ameliorates neuropathic allodynia

**Fig. 2:**
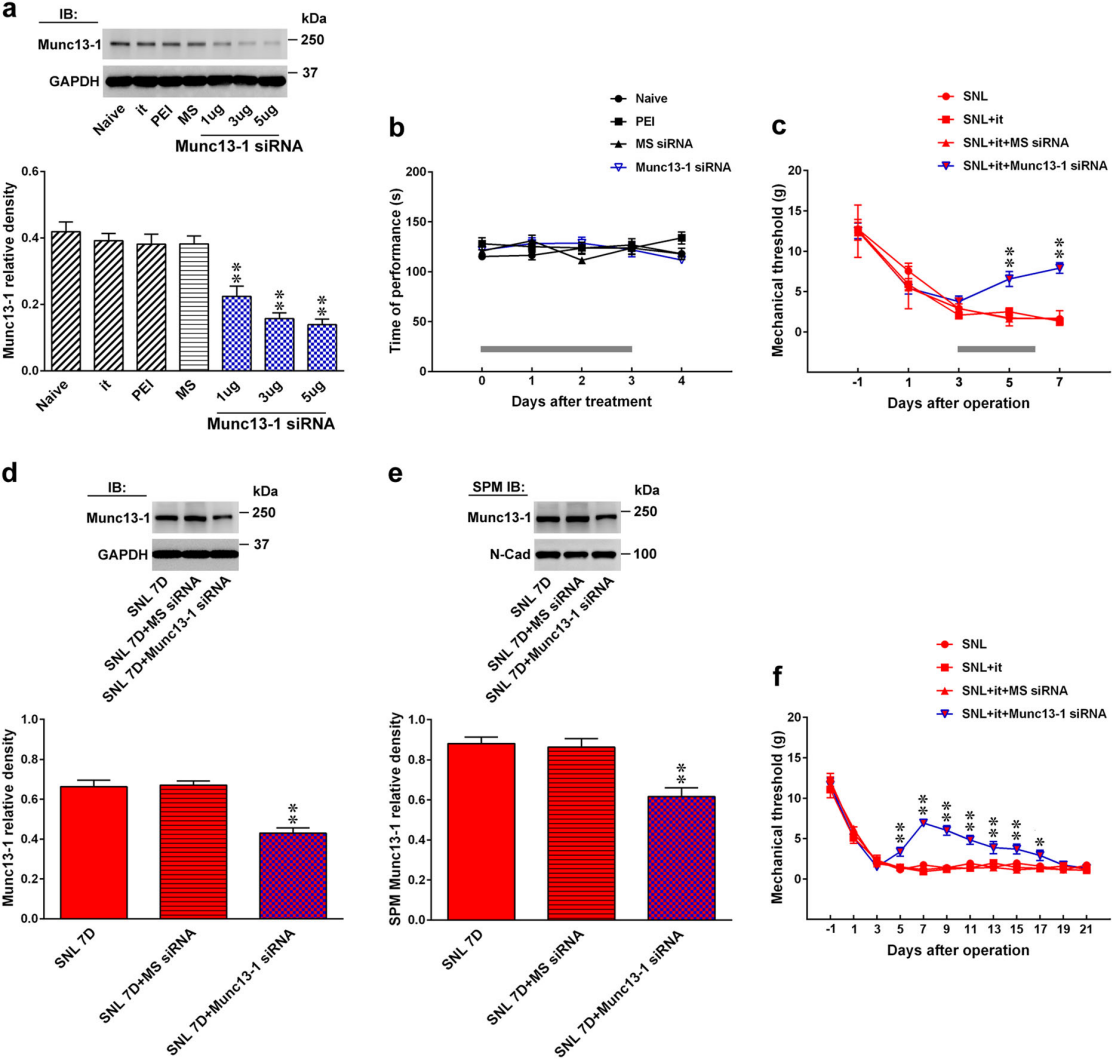
Knockdown of spinal Munc13-1 expression relieves SNL-induced allodynia. **a** Representative Western blot and statistical analysis (normalized to GAPDH) demonstrating that the intrathecal administration of Munc13-1 siRNA (Munc13-1 siRNA; 1, 3, and 5 μg; 10 μL; once daily for 4 days), but not missense siRNA (MS siRNA, 3 μg, 10 μL) or polyethylenimine (a transfection reagent, PEI, 10 μL), dose-dependently decreased spinal Munc13-1 expression in naive rats. it, implantation of an intrathecal catheter. IB immunoblotting. **P < 0.01 compared with naive (*n* = 6). **b** Intrathecal application of neither Munc13-1 siRNA (Munc13-1 siRNAi, 3 μg, 10 μL) nor missense siRNA (MS siRNA, 3 μg, 10 μL) resulted in motor deficits in rats (Rotarod test). The gray bar at the bottom indicates the duration of intrathecal administration (*n* = 7). **c** Intrathecal Munc13-1 siRNA (SNL 7D + it + Munc13-1 siRNA; 3 μg, 10 μL) increased the withdrawal threshold of SNL animals on post-operative days 5 and 7 (von Frey test). The gray bar at the bottom indicates the duration of intrathecal administration. **P < 0.01 compared with SNL (*n* = 7). **d**, **e** Representative Western blot and statistical analysis (normalized to GAPDH and N-Cad, respectively) demonstrating that Munc13-1 siRNA (SNL 7D + Munc13-1 siRNA; 3 μg, 10 μL; once daily at days 3–6 after SNL) decreased the Munc13-1 expression in the total homogenate and synaptic plasma membranes (SPM) of the ipsilateral dorsal horn on post-SNL day 7. IB immunoblotting, SPM IB synaptic plasma membranes immunoblotting. **P < 0.01 compared with SNL 7D (*n* = 6). **f** Treatment of Munc13-1 siRNA (SNL 7D + it + Munc13-1 siRNA; 3 μg, 10 μL; once daily at days 3–6 after SNL) ameliorated the SNL-induced mechanical allodynia from days 5 to 17 after injury. *P < 0.05, **P < 0.01 vs. SNL *n* = 7. Results were expressed as mean ± SEM

To conversely examine the functional relevance of spinal Munc13-1 expression in the development of neuropathic allodynia, we focally knocked down spinal Munc13-1 expression by daily intrathecal injection of small interfering RNA (siRNA) targeted to Munc13-1 mRNA. The results of the Western blot analysis demonstrated that administration with Munc13-1 siRNA (1, 3, and 5 μg; 10 μL; once daily for 4 days), but not missense siRNA (3 μg, 10 μL), polyethylenimine (a transfection reagent, 10 μL), or intrathecal catheter implantation alone, dose-dependently decreased the amount of Munc13-1 in the dorsal horn samples of naive rats (Fig. 2a), which confirmed the efficacy and specificity of the Munc13-1 siRNA. Rotarod analysis revealed that there were no significant differences in the motor performance between the naive and polyethylenimine (10 μL)—missense siRNA (3 μg, 10 μL)—or Munc13-1 siRNA (3 μg, 10 μL)-treated groups (Fig. 2b), indicating that neither our procedures nor the knockdown of spinal Munc13-1 led to motor deficits in the rats. Intriguingly, the von Frey test demonstrated that administering Munc13-1 siRNA (3 μg, 10 μL, once daily from days 3 to 6 after SNL) to SNL rats significantly ameliorated the established allodynia, as evidenced by increasing the withdrawal threshold on days 5 and 7 after operation (Fig. 2c). Moreover, application of Munc13-1 siRNA (3 μg, 10 μL) reversed the enhanced Munc13-1 expression in the total homogenate and the SPM of the ipsilateral dorsal horn dissected at day 7 after SNL (Fig. 2d, e). Additionally, in SNL animals administered Munc13-1 siRNA (3 μg, 10 μL, once daily from days 3 to 6 after SNL), the effect of Munc13-1 siRNA was gradually resolved by day 19 after the operation (Fig. 2f). Collectively, these data provide genetic evidence linking presynaptic Munc13-1 in the dorsal horn to neuropathic injury-induced allodynia.

### Spinal Munc13-1 facilitates presynaptic glutamate release underlying nerve ligation-enhanced dorsal horn neuron excitability

**Fig. 3:**
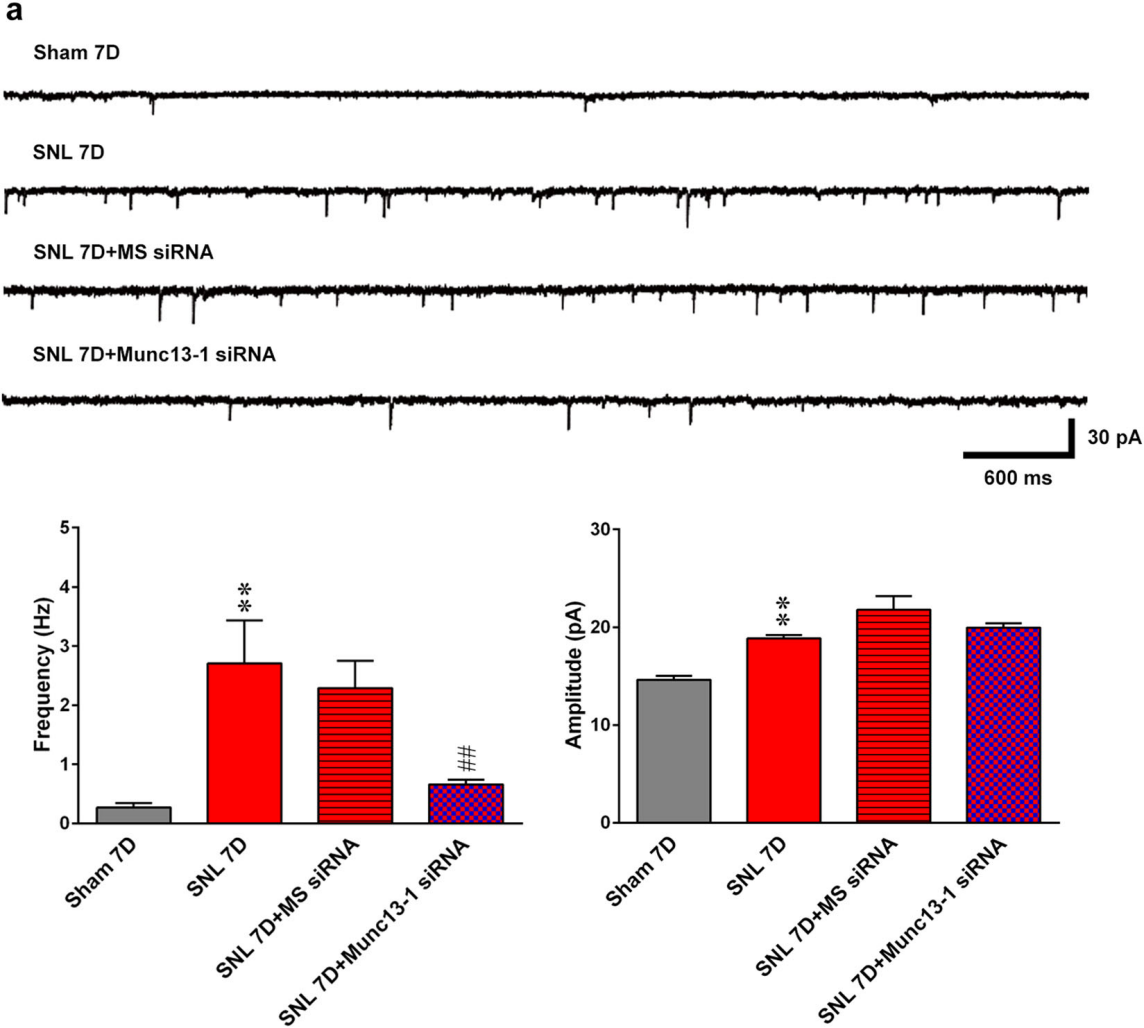
Knockdown of spinal Munc13-1 expression decreases SNL-enhanced mEPSCs frequency in the ipsilateral dorsal horn. **a** Representative traces and statistical analysis of miniature EPSCs (mEPSCs) recorded in the ipsilateral dorsal horn of spinal slices that were dissected from animals receiving the sham operation (Sham 7D) or SNL (SNL 7D) and SNL slices treated with missense siRNA or Munc13-1 siRNA (SNL 7D + MS siRNA or SNL 7D + Munc13-1 siRNA, respectively; 3 μg, 10 μL; once daily at days 3–6 after SNL). The inter-event interval of mEPSCs was significantly shorter and the amplitude was larger in the SNL than in the sham-operated group. Administering Munc13-1 siRNA to SNL rats elongated the inter-event interval without affecting the amplitude of mEPSCs (Kolmogorov–Smirnov test). **P < 0.01 compared with Sham 7D. ^##^*P* < 0.01 compared with SNL 7D (*n* = 4–6). Results were expressed as mean ± SEM

To further confirm the synaptic site where spinal Munc13-1 contributes to the enhanced neuron excitability underlying neuropathic allodynia, we recorded miniature excitatory postsynaptic currents (mEPSCs), which reflect quantal glutamate release from presynaptic terminals^22^, of the ipsilateral dorsal horn neurons in acute spinal slices dissected at day 7 after operation. When compared to the sham operation, SNL significantly increased both the frequency and amplitude of the mEPSCs recorded from dorsal horn neurons at day 7 after operation (Fig. 3a). Notably, administering Munc13-1 siRNA (3 μg, 10 μL) to SNL rats significantly reversed the enhanced frequency but had no effect on the enhanced amplitude of mEPSCs. In contrast, missense siRNA (3 μg, 10 μL) affected neither the SNL-enhanced frequency nor the amplitude of mEPSCs recorded in dorsal horn neurons. This electrophysiological analysis showed that focal knockdown of Munc13-1 expression reversed the SNL-enhanced frequency rather than the amplitude of the mEPSCs, revealing that spinal Munc13-1 contributes to SNL-enhanced excitability of dorsal horn neurons through the presynaptic glutamate release machinery.

### Nerve ligation impedes spinal Fbxo45-dependent Munc13-1 ubiquitination to induce behavioral allodynia

**Fig. 4:**
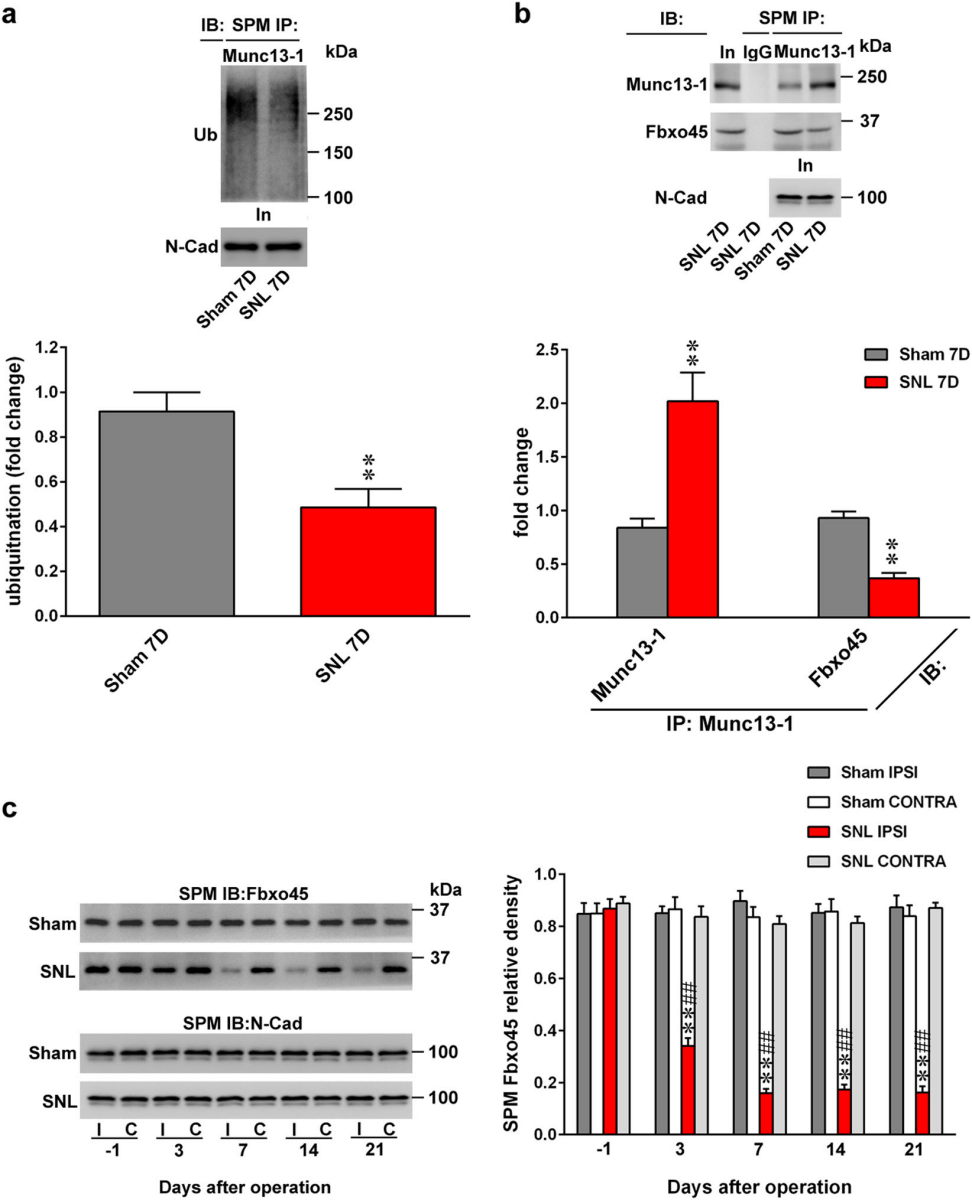
SNL decreases Munc13-1 ubiquitination, Munc13-1–Fbox45 coupling, and Fbxo45 expression in synaptic plasma membranes of dorsal horn. **a** On day 7 after the operation, SNL decreased Munc13-1 ubiquitination in the synaptic plasma membranes (SPM) of the ipsilateral dorsal horn (SNL 7D). SPM IP synaptic plasma membranes immunoprecipitation, IB immunoblotting. Sham 7D, sham operation at day 7. **P < 0.01 compared with Sham 7D (*n* = 5). **b** Immunoprecipitation analysis showing that when compared to sham operation (Sham 7D), SNL (SNL 7D) significantly increased the abundance of Munc13-1-bound Munc13-1 but decreased the abundance of Munc13-1-bound Fbxo45 in the SPM of ipsilateral dorsal horn samples at day 7 after operation. In, input control. **P < 0.01 compared with Sham 7D (*n* = 6). **c** Representative Western blot and statistical analyses (normalized to N-Cad) demonstrating that compared with the sham operation group (Sham), spinal nerve ligation (SNL) in rats decreased Fbxo45 expression in the synaptic plasma membranes of the ipsilateral (I and IPSI), but not the contralateral (C and CONTRA), dorsal horn at days 3, 7, 14, and 21 after operation. **P < 0.01 compared with Sham IPSI. ^##^*P* < 0.01 compared with day −1 (*n* = 6). Results were expressed as mean ± SEM

**Fig. 5:**
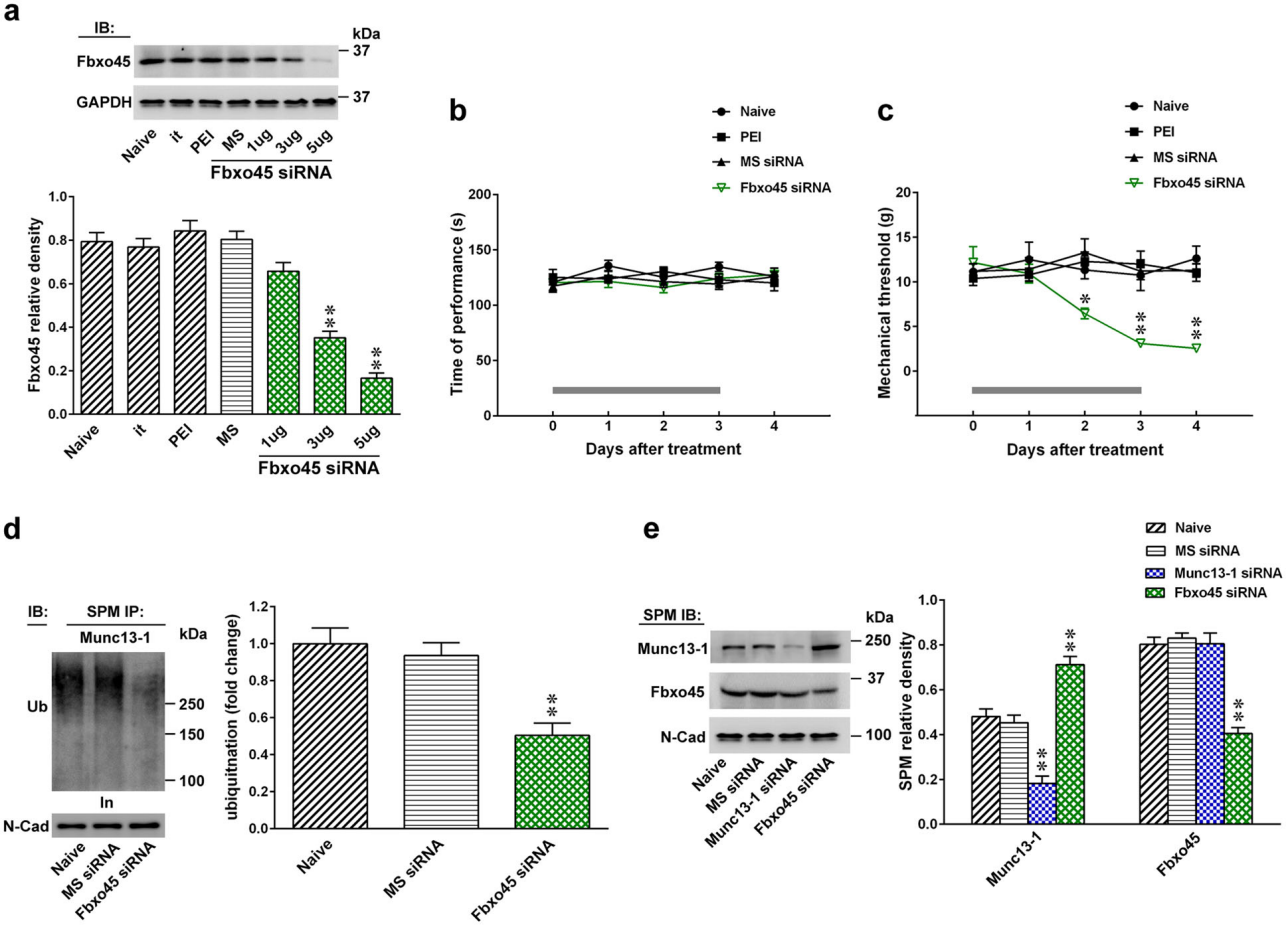
Knockdown of spinal Fbxo45 expression impedes Fbxo45-dependent Munc13-1 ubiquitination to induce behavioral allodynia. **a** Representative Western blot and statistical analysis (normalized to GAPDH) demonstrating that intrathecal administration of Fbxo45 siRNA (Fbxo45 siRNA; 1, 3, and 5 μg; 10 μL; once daily for 4 days), but not missense siRNA (MS siRNA, 3 μg, 10 μL) or polyethylenimine (a transfection reagent, PEI, 10 μL), dose-dependently decreased Fbxo45 expression in the dorsal horn of naive rat. it, implantation of an intrathecal catheter. IB immunoblotting. **P < 0.01 compared with naive (*n* = 6). **b** Intrathecal application of neither Fbxo45 siRNA (Fbxo45 siRNAi, 3 μg, 10 μL) nor missense siRNA (MS siRNA, 3 μg, 10 μL) to naive rats resulted in motor deficits (Rotarod test). The gray bar at the bottom indicates the duration of intrathecal administration (*n* = 7). **c** Intrathecal administration naive rats with Fbxo45 siRNA (Fbxo45 siRNA; 3 μg, 10 μL), decreased the withdrawal threshold of the hind-paw at days 2, 3, and 4 after the start of injection (von Frey test). The gray bar at the bottom indicates the duration of administration. *P < 0.05, **P < 0.01 compared with naive (*n* = 7). **d** The intrathecal application of Fbxo45 siRNA (Fbxo45 siRNAi, 3 μg, 10 μL) into naive rats decreased Munc13-1 ubiquitination in the synaptic plasma membranes of the ipsilateral dorsal horn. SPM IP synaptic plasma membranes immunoprecipitation. Sham 7D, sham operation at day 7. **P < 0.01 compared with naive (*n* = 5). **e** Representative Western blot and statistical analysis (normalized to N-Cad) demonstrating that intrathecal administration naive rats with Munc13-1 siRNA (Munc13-1 siRNA; 3 μg, 10 μL; once daily for 4 days) decreased the Munc13-1 expression without affecting the Fbxo45 expression in the SPM of the ipsilateral dorsal horn. Intrathecal administration naive rats with Fbxo45 siRNA (Fbxo45 siRNA; 3 μg, 10 μL; once daily for 4 days) increased the Munc13-1 expression, but decreased the Fbxo45 expression in the SPM of the ipsilateral dorsal horn. **P < 0.01 compared with naive (*n* = 6). Results were expressed as mean ± SEM

**Fig. 6:**
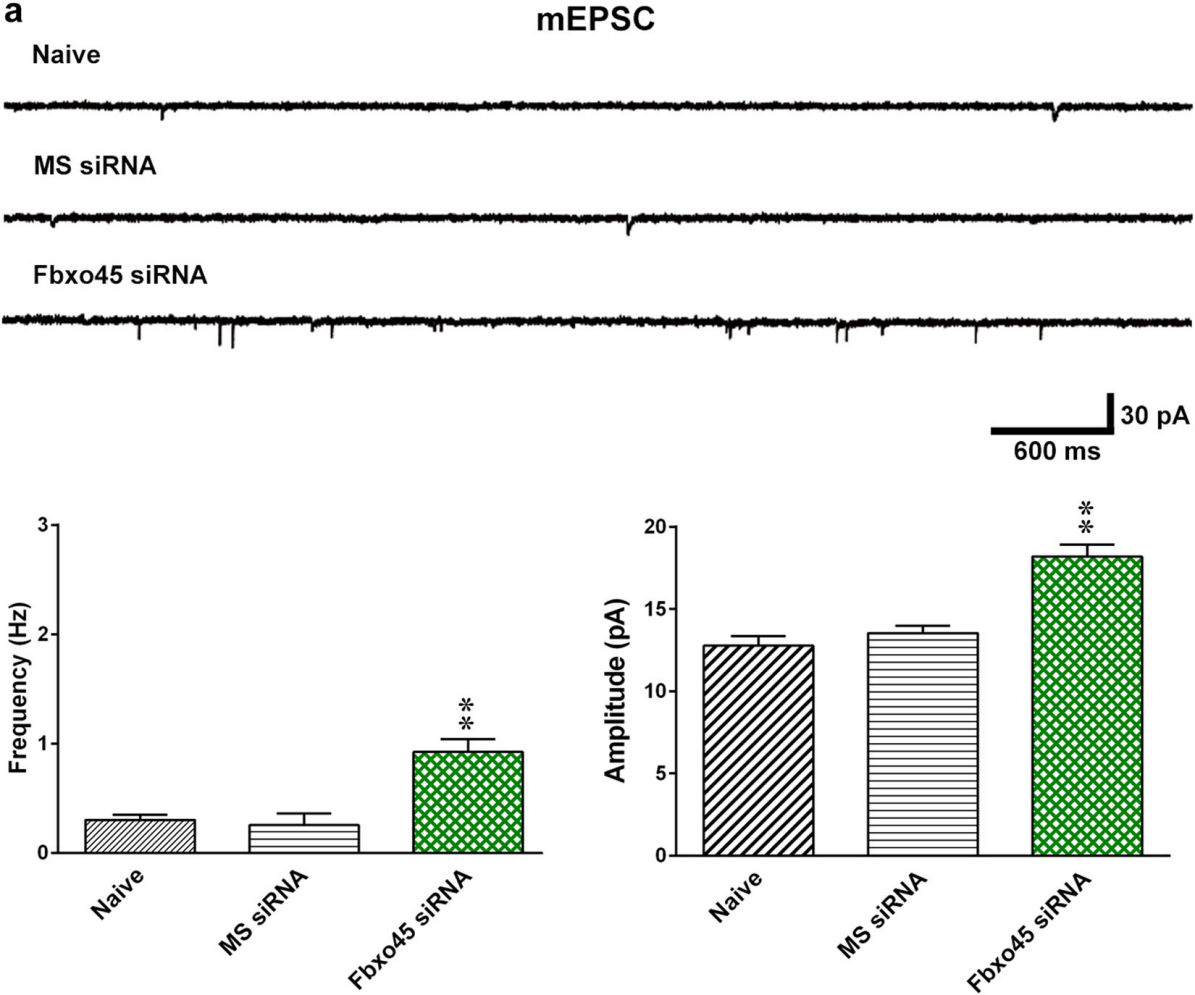
Knockdown of spinal Fbxo45 expression reverses the SNL-enhanced frequency and amplitude of mEPSCs in the ipsilateral dorsal horn. **a** Representative traces and statistical analysis of miniature EPSCs (mEPSCs) recorded in the ipsilateral dorsal horn of spinal slices showing that when compared with naive animals (naive), the inter-event interval of mEPSCs was significantly shorter and the amplitude was larger in animals receiving Fbxo45 siRNA but not missense siRNA (Fbxo45 siRNA and MS siRNA, respectively; 3 μg, 10 μL; once daily for 4 days; Kolmogorov–Smirnov test). **P < 0.01 compared with naive (*n* = 3–4). Results were expressed as mean ± SEM

Ubiquitination-dependent turnover of Munc13-1 critically regulates Munc13-1 expression in neurons^23,24^. Thus, we first investigated the potential contribution of ubiquitination-dependent spinal Munc13-1 in neuropathic pain development. At day 7 post surgery, SNL predictably decreased Munc13-1 ubiquitination in the ipsilateral dorsal horn (Fig. 4a). Based on this result and considering that Fbxo45, a novel synaptic E3 ubiquitin ligase, was shown to impact neural plasticity-associated transmission by modifying synaptic Munc13-1 ubiquitination^13^, we hence tested whether the neuropathic injury enhances presynaptic Munc13-1 expression in the dorsal horn to underlie behavioral allodynia through impeding Fbxo45-dependent Munc13-1 ubiquitination. The results of the co-precipitation analysis demonstrated that concomitant with decreasing Munc13-1 ubiquitination, SNL increased Munc13-1–Munc13-1 interaction and inhibited the Munc13-1–Fbxo45 interaction at the SPM of the ipsilateral dorsal horn samples at day 7 after operation compared with the sham operation (Fig. 4b). Moreover, SNL selectively decreased the amount of Fbxo45 at the SPM of the ipsilateral, but not contralateral, dorsal horn on days 3, 7, 14, and 21 after operation (Fig. 4c), which temporally correlated with the SNL-induced allodynia and Munc13-1 expression. To provide further genetic basis supporting the role of Fbxo45-dependent Munc13-1 ubiquitination in the development of neuropathic allodynia, we focally knocked down spinal Fbxo45 expression by daily intrathecal administering animals with Fbxo45 siRNA. Our procedure effectively knocked down spinal Fbxo45 expression (Fig. 5a) but did not result in motor deficits (Fig. 5b) because Fbxo45 siRNA (1, 3, and 5 μg; 10 μL; once daily for 4 days) dose-dependently decreased the abundance of Fbxo45 in dorsal horn samples but exhibited no effect on the performance time measured by the Rotarod test (3 μg, 10 μL). Notably, administering Fbxo45 siRNA (3 μg; 10 μL) to naive rats not only triggered behavioral allodynia—as evidenced by a significant decrease in the withdrawal threshold of the hind-paw at days 2, 3, and 4 (Fig. 5c)—but also decreased Munc13-1 ubiquitination in the SPM of dorsal horn samples rats at day 4 after the starting of treatment (Fig. 5d). In contrast to Fbxo45 siRNA (3 μg, 10 μL)-mediated decreased Fbxo45 expression and increased Munc13-1 expression, administering Munc13-1 siRNA (3 μg, 10 μL) to naive rats reduced the expression of Munc13-1 without affecting Fbxo45 expression in the SPM of dorsal horn dissected at day 4 after the starting of treatment (Fig. 5e). Surprisingly, electrophysiological recordings of dorsal horn slices demonstrated that administering Fbxo45 siRNA (3 μg, 10 μL) to naive animals significantly increased both the frequency and amplitude of the mEPSCs (Fig. 6a). Collectively, these data provide evidence supporting the role of Fbxo45-dependent ubiquitination in the spinal machinery underlying neuropathic allodynia by possibly modifying the turnover of Munc13-1.

### Nerve ligation induces allodynia via TNF-α-impeded spinal Fbxo45-dependent Munc13-1 ubiquitination

**Fig. 7:**
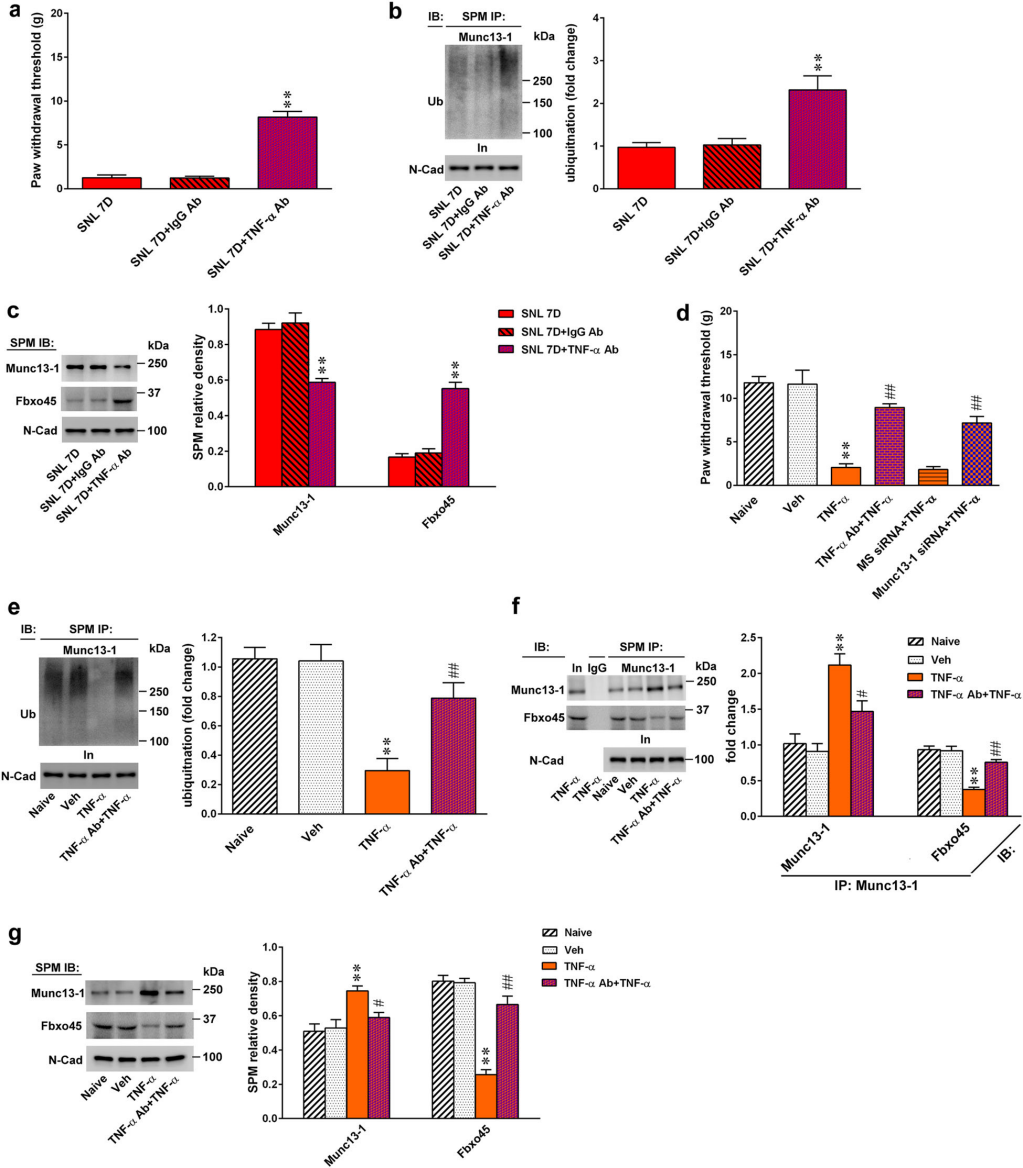
Nerve ligation induces allodynia via TNF-α-impeded spinal Fbxo45-dependent Munc13-1 ubiquitination. **a** Intrathecal application of TNF-α-neutralizing antibody (SNL 7D + TNF-α Ab; 100 ng, 10 μL) to SNL rats significantly increased the withdrawal threshold (von Frey test). **P < 0.01 compared with SNL 7D (*n* = 7). **b** Intrathecal application of TNF-α-neutralizing antibody (SNL 7D + TNF-α Ab; 100 ng, 10 μL) to SNL rats increased Munc13-1 ubiquitination in the synaptic plasma membrane (SPM) of the ipsilateral dorsal horn. In input control, SPM IP synaptic plasma membrane immunoprecipitation, IB immunoblotting. **P < 0.01 compared with SNL 7D (*n* = 5). **c** Representative Western blot and statistical analysis (normalized to N-Cad) demonstrating that intrathecal application of TNF-α-neutralizing antibody (SNL 7D + TNF-α Ab; 100 ng, 10 μL) to SNL rats decreased Munc13-1 expression but increased the Fbxo45 expression in the SPM of the ipsilateral dorsal horn. **P < 0.01 compared with SNL 7D (*n* = 6). **d** Intrathecal application of TNF-α (TNF-α, 1 pM, 10 μL) to naive rats significantly decreased the withdrawal threshold at 2 h post injection, which was markedly reversed by pretreating animals with TNF-α-neutralizing antibody (TNF-α + TNF-α Ab; 100 ng, 10 μL) and Munc13-1 siRNA (Munc13-1 siRNA + TNF-α; 3 μg, 10 μL; once daily for 4 days) (von Frey test). **P < 0.01 compared with naive. ^##^*P* < 0.01 compared with TNF-α (*n* = 7). **e** Intrathecal injection of TNF-α (TNF-α, 1 pM, 10 μL) into naive animals decreased Munc13-1 ubiquitination in the SPM of the ipsilateral dorsal horn, which was markedly reversed by pretreatment with TNF-α-neutralizing antibody (TNF-α Ab + TNF-α, 100 ng, 10 μL). **P < 0.01 compared with naive. ^##^*P* < 0.01 compared with TNF-α (*n* = 5). **f** Immunoprecipitation showing that spinal TNF-α (TNF-α, 1 pM, 10 μL) injection to naive rats significantly increased the abundance of Munc13-1-bound Munc13-1 but decreased the abundance of Munc13-1-bound Fbxo45 in the SPM of ipsilateral dorsal horn, which were markedly reversed by pretreating animals with TNF-α-neutralizing antibody (TNF-α Ab + TNF-α, 100 ng, 10 μL). In input control. **P < 0.01 compared with naive. ^#^*P* < 0.05, ^##^*P* < 0.01 compared with TNF-α. **g** Representative Western blot and statistical analysis (normalized to N-Cad) demonstrating that intrathecal injection of TNF-α (TNF-α, 1 pM, 10 μL) into naive rats increased Munc13-1 expression but decreased Fbxo45 expression in the SPM of the ipsilateral dorsal horn, which was markedly reversed by spinal pretreatment with TNF-α-neutralizing antibody (TNF-α Ab + TNF-α, 100 ng, 10 μL). **P < 0.01 compared with naive. ^#^*P* < 0.05, ^##^*P* < 0.01 compared with TNF-α (*n* = 6). Results were expressed as mean ± SEM

**Fig. 8:**
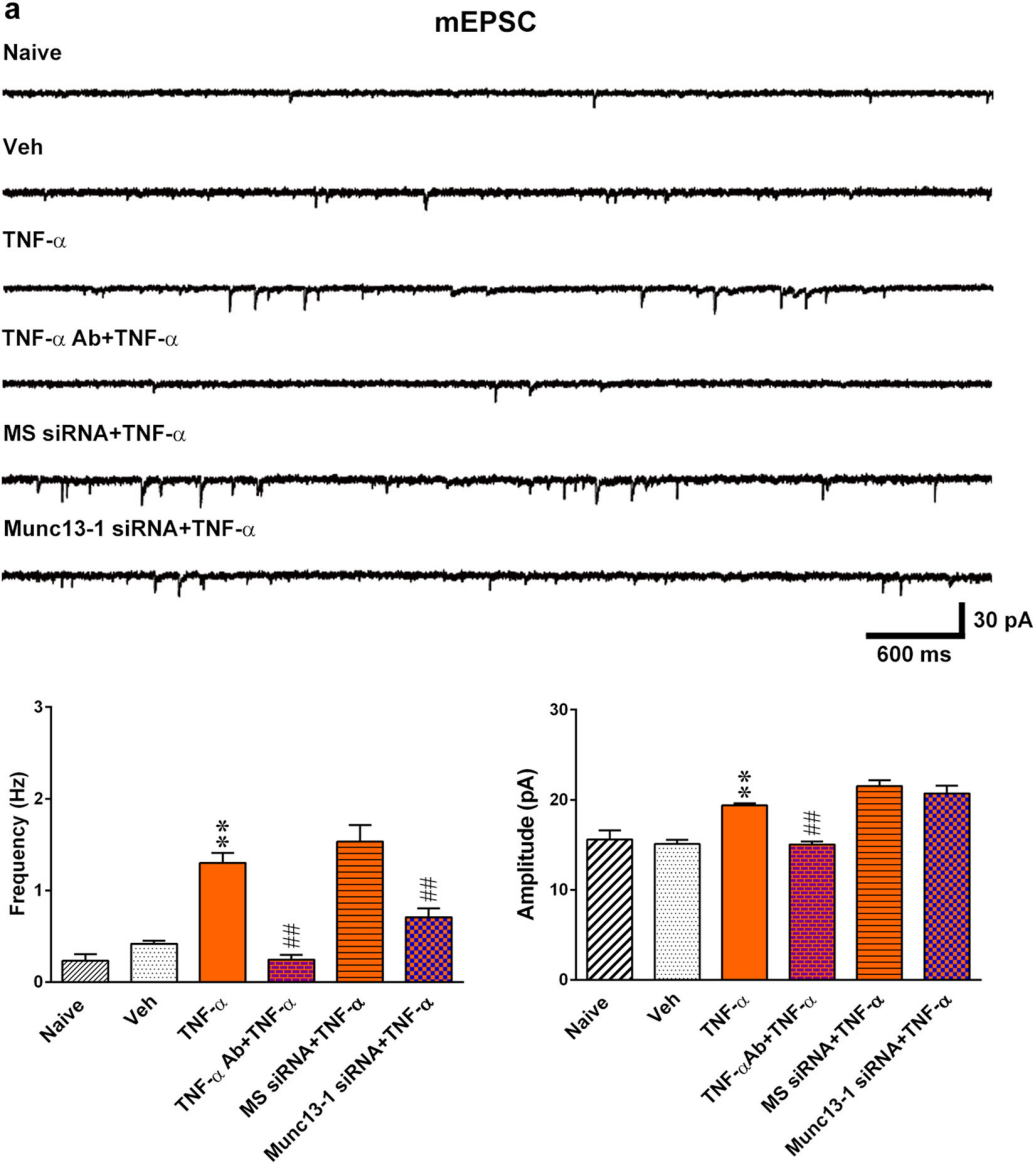
TNF-α increased both the frequency and amplitude of the mEPSCs recorded from dorsal horn neurons. **a** Representative traces and statistical analysis of miniature EPSCs (mEPSCs) recorded in the ipsilateral dorsal horn of spinal slices showing that spinal administration of TNF-α to naive (naive) rats (TNF-α, 1 pM, 10 μL) increased both the frequency and amplitude of the mEPSCs. Pretreatment with TNF-α-neutralizing antibody (TNF-α Ab + TNF-α; 100 ng, 10 μL) reversed the TNF-α-increased frequency and amplitude, but Munc13-1 siRNA (Munc13-1 siRNA + TNF-α; 3 μg, 10 μL; once daily for 4 days) reversed the TNF-α-increased frequency without affecting the TNF-α-increased amplitude (Kolmogorov–Smirnov test). **P < 0.01 compared with naive. ^##^*P* < 0.01 compared with TNF-α (*n* = 4–6). Results were expressed as mean ± SEM

Because our previous publication demonstrated that TNF-α contributes to neuropathic pain development by targeting spinal F-box protein-associated ubiquitination^20^, we next examined whether spinal TNF-α participates in neuropathic pain development via modifying Fbxo45-dependent Munc13-1 ubiquitination. First, we examined whether antagonizing spinal TNF-α affects SNL-induced allodynia. At day 7 post SNL, intrathecal injections of a TNF-α-neutralizing antibody (100 ng, 10 μL), but not a non-specific IgG (100 ng, 10 μL), increased the withdrawal threshold of the ipsilateral hind-paw (Fig. 7a). Next, intrathecal injections of a TNF-α-neutralizing antibody (SNL 7D + TNF-α Ab; 100 ng, 10 μL) increased the degree of Munc13-1 ubiquitination (Fig. 7b), decreased the abundance of Munc13-1 protein, and increased the abundance of Fbxo45 protein (Fig. 7c) at day 7 post-SNL. To further confirm the role of spinal TNF-α in the neuropathic pain-associated, Fbxo45-dependent, spinal Munc13-1 ubiquitination pathway, recombinant rat TNF-α was intrathecally administered to naive rats. Two hours after spinal injection, TNF-α (1 pM, 10 μL) was found to trigger allodynia in naive rats, as evidenced by a significant decrease in the paw withdrawal threshold (Fig. 7d), and this effect was prevented by pretreating animals with a bolus of TNF-α-neutralizing antibody (100 ng, 10 μL) and daily Munc13-1 siRNA (3 μg; 10 μL). Furthermore, 2 h after application, TNF-α (1 pM, 10 μL) significantly decreased the degree of Munc13-1 ubiquitination, Fbxo45-Munc13-1 co-precipitation, and Fbxo45 protein expression levels but increased Munc13-1 protein expression and Munc13-1–Munc13-1 co-precipitation; these effects were reversed by spinal injection of TNF-α-neutralizing antibody (100 ng, 10 μL; Fig. 7e–g). Electrophysiological analyses demonstrated that spinal application of TNF-α (1 pM, 10 μL) increased both the frequency and amplitude of the mEPSCs recorded from dorsal horn neurons (Fig. 8a), which were reversed by TNF-α-neutralizing antibody (100 ng, 10 μL). In contrast, focal knockdown of spinal Munc13-1 expression using daily injection of Munc13-1 siRNA (3 μg; 10 μL) reversed the TNF-α-enhanced frequency but not the amplitude of the mEPSCs in dorsal horn neurons. Collectively, these results provided a pharmacological basis that supports the crucial role of TNF-α in SNL-induced neuropathic pain, potentially via impeding Fbxo45-mediated Munc13-1 ubiquitination and degradation.

## Discussion

The role of the active zone protein Munc13-1 in spinal plasticity underlying neuropathic pain remains unknown. In the current study, we tested the role of spinal Munc13-1 in the development of neuropathic pain. Mechanistically, we demonstrated that spinal TNF-α contributes to neuropathic allodynia by impeding Fbxo45-dependent Munc13-1 ubiquitination and degradation, and thus this accumulation of Munc13-1 in the presynaptic area facilitates synaptic excitability of dorsal horn neurons and underlies the development of behavioral allodynia.

Neurotransmitter release is one of the elementary components of synaptic transmission in the nervous system. By organizing vesicle docking and priming, active zone proteins mediate activity-dependent modification of vesicle release that underlies forms of plastic changes in synaptic function^2^. Munc13, a highly interesting active zone protein, plays a crucial role in synaptic vesicle priming machinery, such that genetic elimination of Munc13 expression severely disrupts synaptic transmission in vertebrates^25,26^. In the Munc13 family, Munc13-2 is recognized as being present in only rostral brain regions such as the cerebral cortex^27^, Munc13-3 expression is mostly restricted to the cerebellum^27^, and Munc13-4 is predominantly expressed in the lung^28^. Unlike other Munc13 isoforms that exhibit strikingly differential expression patterns, Munc13-1 is expressed in all neurons of the rat central nervous system^28^. Although the glutamatergic hippocampal neurons of mice form ultrastructurally normal synapses, mutation of Munc13-1 arrests the synaptic vesicle cycle at the maturation step, revealing a role of Munc13-1 in regulating excitatory glutamatergic neurotransmission^29^. Consistent with evidence demonstrating that glutamatergic synaptic transmission at the dorsal horn pivotally contributes to pain sensitization caused by neuropathic insults^30^, our findings in the present identify a role of spinal Munc13-1 in pain-associated plasticity by impacting glutamatergic neurotransmission because experimental neuropathic injury enhanced frequency of mEPSCs, an index reflecting quantal glutamate release;^31^ effect that can be reversed by genetic knockdown of spinal Munc13-1 expression.

After proteasome inhibition, a Munc13-1 homolog was found to selectively accumulate in the presynaptic terminals of the neuromuscular junction in Drosophila^9,11^. In rat hippocampal slices, pharmacological antagonism of the ubiquitin–proteasome system using a wide-spectrum inhibitor prevented the induction of persistent presynaptic silencing associated with decreasing Munc13-1 levels^24^. Based on our previous publication that revealed a complicated ubiquitination-associated regulation of an active zone protein RIM1α by showing that Fbxo3-dependent Fbxl2 ubiquitination modifies Fbxl2-dependent RIM1α ubiquitination^7^, we speculate that application of wide-spectrum protease inhibitor, such as a pan ubiquitination inhibitors MG-132^32,33^ might have multiple effects on ubiquitination pathways. Therefore, unlike these studies pharmacologically antagonizing Munc13-1 ubiquitination using a wide-spectrum protease inhibitor, we investigated Fbxo45-dependent Munc13-1 ubiquitination by focal knockdown of spinal Fbxo45 expression. The results in this study showed that knockdown of spinal Fbxo45 expression decreased Munc13-1 ubiquitination and that Munc13-1 accumulates in the dorsal horn, revealing a role of Fbxo45-dependent ubiquitination machinery in spinal Munc13-1 turnover. Moreover, using an animal model mimicking neuropathic injury, we found that SNL impeded Fbxo45-dependent Munc13-1 degradation, which enhanced Munc13-1 expression in the dorsal horn and thus resulted in the development of pain-associated plasticity. These findings are consistent with a previous study demonstrating that Fbxo45 induces Munc13-1 degradation and that Munc13-1 serves as a target or downstream molecule of Fbxo45 at the synaptic site^13^. Our findings identified Fbxo45 as a regulator of spinal Munc13-1 ubiquitination post-neuropathic injury and hence suggested a novel strategy for the development of medical strategies for pain relief. In addition, we examined the ubiquitination-associated Munc13-1 degradation by focusing on spinal Fbxo45 expression. However, because one of our previous studies identified that two E3 ligase isoforms, Fbxo3 and Fbxl2, work co-operatively as a part of the spinal active zone protein ubiquitination machinery that impacts spinal plasticity underlying neuropathic pain development^7^, the potential role of other E3 ligases in pain-associated Munc13-1 turnover in the dorsal horn requires further investigation.

In the present study, it is worth noting that although SNL enhanced both the frequency and amplitude of mEPSCs recorded from dorsal horn neurons, knockdown of spinal Munc13-1 expression reversed the SNL-enhanced frequency, but not the amplitude, of mEPSCs. This result suggests that Munc13-1 impacts pain-associated spinal plasticity via a presynaptic glutamatergic machinery, which is consistent with studies showing that the expression of members of the Munc protein family are spatially restricted to the presynaptic active zones^23^. Nevertheless, in this study, knockdown of spinal Fbxo45 not only enhanced the mEPSCs frequency but also reversed the SNL-enhanced amplitude of the mEPSCs in the dorsal horn neurons, which was evidenced by the finding that Fbxo45-dependent allodynia relied on presynaptic and postsynaptic glutamatergic neural transmission in the dorsal horn. Our conclusion is supported by the study of Tada et al. in which histochemical analysis demonstrated that Fbxo45 partially colocalized with a presynaptic marker VGlut1 and a postsynaptic marker PSD-95^13^. Our laboratory previously found that Fbxo3 contributes to neuropathic pain development through the ubiquitination machinery by impacting the postsynaptic glutamatergic neurotransmission and glutamate receptor 1 (GluR1) trafficking^20^. In the present study, we found that Fbxo45-dependent ubiquitination underlies pain-associated plasticity by modifying postsynaptic substrates to affect spinal glutamatergic signaling; however, the potential involvement of spinal GluR1 trafficking in Fbxo45-modified glutamatergic transmission remains to be further investigated. Moreover, it will be very interesting to identify potential candidates downstream of Fbxo45 in mediating pain-associated spinal plasticity.

Recently, evidence has shown that active zone components Munc13-1 and RIM1, which are putative vesicle priming and tethering proteins, respectively, functionally interact at the synaptic site and that disruption of the Munc13-1–RIM1α interaction results in a loss of fusion-competent synaptic vesicles^1^. The interaction between RIM1α and Munc13-1 has been recently linked to presynaptic long-term plasticity at mossy fiber synapses^23^. Considering that RIM1α is a protease substrate^12^ and that our previous publication demonstrated E3 ligase-dependent RIM1α ubiquitination crucially contributes to the development of neuropathic pain^7^, the possibility that RIM1α and Munc13-1 ubiquitination work synergistically to mediate spinal synaptic plasticity underlying neuropathic pain requires further investigation.

Nerve injury-induced release of TNF-α, a well-known inflammatory cytokine, has been proposed to be an initiator of neuropathic pain^18^, and conversely, inhibition of TNF-α release reduces pain in SNL rats^34^. Notably, in Parkin-deficient cells, the degree of protein ubiquitination is significantly reduced after TNF-α application^35^, and our previous study revealed that spinal TNF-α contributes to the development of neuropathic pain by impacting F-box protein-dependent ubiquitination^20^. Consistent with these observations linking TNF-α-associated ubiquitination to pain hypersensitivity, the results in the current study demonstrated that spinal application of a TNF-α-neutralizing antibody ameliorated the SNL-induced allodynia that accompanied Fbxo45 expression and Munc13-1 ubiquitination, which decreased Munc13-1 expression in the dorsal horn. Moreover, spinal TNF-α injection significantly decreased the degree of Munc13 ubiquitination, Fbxo45-Munc13-1 interaction, and Fbxo45 expression but increased Munc13-1 expression, and these effects were all reversed by administering a TNF-α-neutralizing antibody to the animals. Our findings identify spinal TNF-α as an initiator that triggers experimental neuropathic injury-induced behavioral allodynia by impacting Fbxo45-mediated Munc13-1 ubiquitination/degradation and thus could provide new insights into the use of TNF-α-triggered protein ubiquitination/degradation in the dorsal horn as an important strategy in pain research. Nevertheless, the extent of recovery in the paw withdrawal threshold produced using the TNF-α antibody is far below the baseline of naive rat, and clinical trials with monoclonal TNFα antibodies have largely failed to show any effect on radicular pain. Moreover, during the acute phase of inflammation, the expression levels of pro-inflammatory cytokines, such as IL-1β, 1L-6, and TNF-α, are elevated to facilitate the synthesis and release of positive acute-phase proteins that accelerate and augment inflammation^36,37^. In the present study, although we investigated the development of neuropathic pain by focusing on the role of TNF-α, other pro-inflammatory cytokines, such as IL-1β and 1L-6, are elevated in various neuropathic pain conditions^38,39^, and the anti-inflammatory cytokine IL-10 efficiently reduces neuropathic pain by downregulating the expression of TNF-α, IL-1β, and IL-6^40^. Notably, in the central nervous system, the production of TNF-α, IL-1β, and IL-6 from glial cells plays a critical role in processing abnormal pain signaling^41^. Hence, the potential contribution of cytokines other than TNF-α should be further examined to elucidate whether the development of neuropathic pain involves complex cytokine-mediated neuron–glia interactions.

## Materials and methods

### Animal preparations

Adult male Sprague–Dawley rats weighing 200–250 g were used throughout this study. They were housed at room temperature (23 + 1 °C) with a 12-h light–dark cycle (lights on 8:00 a.m.–8:00 p.m.) and fed food and water ad libitum. The surgical procedure and experimental protocols performed in this study were conducted in accordance with the guidelines of the International Association for the Study of Pain^42^ and were reviewed and approved by the Institutional Review Board of Taipei Medical University, Taipei, Taiwan. Animals were randomly allocated to treatment groups using a Research Randomizer, a randomizer on the website “https://www.randomizer.org/”, and the sample size of each group was based on our previous experience. In each group, seven rats were used for behavioral testing and immunohistochemistry; six rats were used for Western blot analysis, ubiquitination studies, and co-precipitation studies; and three–six rats were used for electrophysiological analysis. All the investigators were blinded to the treatment groups for all experiments.

### Spinal nerve ligation

SNL, which mimics neuropathic injury, was carried out as protocol described elsewhere^7,20,43,44^. Briefly, the rats were anesthetized with isoflurane (induction, 5%; maintenance, 2% in air, Baxter, Guayama, Puerto Rico, USA). After an incision was made, the left L5 and L6 spinal nerves were carefully isolated from the surrounding tissue and then tightly ligated with 6–0 silk sutures. Finally, the wound and the surrounding skin were sutured. In the sham operation group, the surgical procedures were identical to the nerve-ligated animals, except the silk sutures were left unligated.

### Intrathecal catheter

Implantation of intrathecal cannula was performed as described in our previous studies^20,45,46^. In short, a PE-10 silastic tubing was implanted in the lumbar enlargement of the spinal cord under isoflurane anesthetized (induction, 5%; maintenance, 2% in air). The outer part of the catheter was plugged and immobilized onto the skin on closure of the wound. After 3 days of recovery, animal with any sign of neurological deficits were discarded and excluded from further experiments.

### Behavioral studies

To assess the development of mechanical allodynia, rats were individually placed in an opaque plastic cylinder, which was placed on a wire mesh and habituated for 1 h to allow acclimatization to the environment. Von Frey monofilaments (Stoelting, Wood Dale, IL) were then applied to the plantar surface of the hind-paws of animals to measure the paw withdrawal threshold (up-down method) according to a modification of a previously described method^47^. After acclimatization, the calibrated von Frey filaments, with logarithmically incremental stiffness from 0.6 to 26.0 g, were used to measure paw withdrawal. Beginning with a 10 g probe, the filament was vertically applied to the plantar surface of the rat paw for 4 s while the filament was bent. Each trial of stimuli consisted of five applications of the filament every 4 s, and brisk foot withdrawals at least three out of the five times the filament was applied were considered positive, with a lack of response considered negative. If a positive response was observed, the filament with the next lowest stiffness was applied; otherwise, the filament with the next highest stiffness was used. The stimulus force that produced a 50% likelihood of paw withdrawal was determined as the withdrawal threshold using the Dixon “up-down” method. The motor function of animals was assessed in a rotarod apparatus (Panlab Harvard Apparatus, Barcelona, Spain). For acclimatization, the animals were subjected to three training trials at 3–4 h intervals on 2 separate days. During the training sessions, the rod was set to accelerate from 4 to 30 rpm over a 180 s period. During the test session, the performance times of the rats were recorded until the cut-off time of 180 s. Three measurements were obtained at 5 min intervals and averaged for each test.

### Subcellular fractionation

After animals were euthanized, laminectomy (L3–L6) was performed to expose the spinal cord. The lumbar enlargement of the spinal cord (L4–L5) was the dissected, and then the dorsal quadrants were removed and homogenized in 25 mM Tris-HCl, 150 mM NaCl, 1% NP-40, 1% sodium deoxycholate, and 0.1% SDS supplemented with a complete protease inhibitor mixture (Roche, Upper Bavaria, Germany). After incubation on ice (1 h), the lysates were centrifuged (14,000 rpm, 20 min, 4 °C). The supernatant was recognized as total homogenate. Subcellular fractions were prepared and modified according to a previously described method^48^. Briefly, the dorsal horn samples were homogenized in ice-cold sucrose/HEPES buffer (0.32 M sucrose, 10 mM HEPES, pH 7.4) containing protease inhibitors and phosphatase inhibitor and were centrifuged at 800 g, 10 min. The supernatant (S1) was separated from the pellet (P1), which contained nuclei and debris. The collected supernatant (S1) was centrifuged at 9000 × *g* for 20 min to obtain supernatant (S2) and a crude synaptosomal fraction from the pellet (P2). P2 was washed once in sucrose/HEPES buffer, hypotonically lysed and centrifuged at 25,000 × *g* for 20 min to isolate synaptosomal membranes from the pellet (LP1), whereas the free synaptic vesicles remained in the supernatant (LS1). LP1 was resuspended with 1.1 M sucrose/HEPES buffer, layered on the bottom of a discontinuous sucrose gradient (0.855 and 0.32 M) and centrifuged for 2.5 h at 19,000 rpm, thus resulting in the isolation of myelin (in the 0.32/0.855 M sucrose interface), SPMs (in the 0.855/1.1 M sucrose interface), and mitochondria (in the pellet). All protein concentrations were determined with a BCA protein assay kit (Pierce, Rockford, IL), using BSA as a standard.

### Western blot analysis

As described above, the dorsal quadrants of the spinal cord (L4–5) of animals were dissected after euthanization. Samples were homogenized in 25 mM Tris-HCl, 150 mM NaCl, 1% NP-40, 1% sodium deoxycholate, and 0.1% SDS supplemented with a complete protease inhibitor mixture (Roche, Upper Bavaria, Germany). After incubation on ice (1 h), the lysates were centrifuged (14,000 rpm, 20 min, 4 °C) and the supernatant were collected. All protein concentrations were determined using a BCA protein assay reagent kit (Pierce, Rockford, IL, USA). Briefly, equal amounts of samples were separated by SDS-PAGE and electrophoretically transferred to PVDF membranes. The membranes were blocked with 5% non-fat milk or BSA in TBS containing 0.1% Tween-20 for 1 h and then incubated with primary antibodies at 4 °C for 1 h. The primary antibodies were as follows: anti-Munc13–1 (rabbit, 1:1000, Synaptic Systems, Goettingen, Germany), anti-Fbxo45 (rabbit, 1:1000, bioss, Woburn, MA, USA), anti-N-cadherin (mouse, 1:2000, Thermo Scientific, Rockford, IL), and anti-GAPDH (mouse, 1:2000, Santa Cruz Biotechnology, Santa Cruz, CA, USA). Then, the blots were washed and incubated (1 h, room temperature) with peroxidase-conjugated goat anti-rabbit IgG (1:8000, Jackson ImmunoResearch, West Grove, PA, USA) or goat anti-mouse IgG (1:8000, Jackson ImmunoResearch) antibodies. The protein bands were visualized using an enhanced chemiluminescence detection kit (ECL Plus, Millipore) and then subjected to densitometric analysis using Science Lab 2003 software (Fuji, Tokyo, Japan).

### Co-precipitation studies

The dorsal quadrants of the spinal cord (L4–5) of animals were dissected after euthanization as described above. Co-precipitation of dorsal horn was performed as previously described^49,50^. Briefly, extractions of dorsal horn samples were incubated with a rabbit polyclonal antibody against Munc13-1 (rabbit, 1:1000, Synaptic Systems, Goettingen, Germany) overnight at 4 °C. At 1:1 slurry protein agarose suspension (Millipore) was added to the protein immunocomplex, and the mixture was incubated at 4 °C for 2–3 h. Agarose beads were washed once with 1% (v/v) Triton X-100 in the immunoprecipitation buffer (50 mM Tris-HCl, pH 7.4, 5 mM EDTA, 0.02% (w/v) sodium azide), twice with 1% (v/v) Triton X-100 in immunoprecipitation buffer plus 300 mM NaCl, and three times with only immunoprecipitation buffer. The bound proteins were eluted in SDS-PAGE sample buffer at 95 °C. The proteins were then separated on SDS-PAGE, electrophoretically transferred to polyvinylidene difluoride membranes and detected using anti-Munc13-1 (rabbit, 1:1000, Synaptic Systems, Goettingen, Germany), anti-Fbxo45 (rabbit, 1:1000, bioss, Woburn, MA, USA), or mouse anti-ubiquitin (1:1000; FK2; Enzo Life Sciences, Madison Avenue, USA).

### Immunofluorescence analysis

Rats were deeply anesthetized and perfused intracardially with PBS followed by 4% paraformaldehyde/PBS (pH 7.4). The dorsal horn (L4–5) samples were removed, post-fixed in the same fixative (4 °C for 4 h) and cryoprotected in 30% sucrose solution for overnight at 4 °C. Transverse lumbar spinal cord sections (30 µm) were cut using a cryostat and mounted on glass slides. The sections were pre-incubated with 5% BSA 1 h in PBS to block non-specific binding. Subsequently, the sections were incubated with rabbit anti-Munc13-1 (rabbit, 1:200, Synaptic Systems, Goettingen, Germany), together with mouse anti-synaptophysin (a presynaptic marker, 1:500, Abcam, Cambridge, USA), or mouse anti-homer1 (1:500, Abcam, Cambridge, USA) overnight (4 °C). After three times of rinsing with PBS, spinal sections were incubated at 37 °C for 1 h with Alexa Fluor 594-conjugated goat anti-rabbit IgG (1:1500; Invitrogen, Grand Island, NY) as well as with Alexa Fluor 488-conjugated goat anti-mouse IgG (1:1500; Invitrogen, Grand Island, NY). The spinal sections were subsequently rinsed in PBS, and coverslips were applied. When these fluorescent markers were excited, they were easily detected by a camera-coupled device (X-plorer; Diagnostic Instruments, Inc., USA) through a fluorescent microscope (LEICA DM2500, Germany).

### Spinal slice preparations

Under anesthesia with isoflurane, rats were underwent laminectomy for removal of the lumbar spinal cord. The lumbar spinal cord was placed in ice-cold sucrose artificial cerebrospinal fluid (aCSF) bubbled with 95% O_2_/5% CO_2_. The sucrose aCSF consisted of the following (in mM): 234 sucrose, 3.6 KCl, 1.2 MgCl_2_, 2.5 CaCl_2_, 1.2 NaH_2_PO_4_, 12 glucose, and 25 NaHCO_3_. The lumbar spinal cord slices (300 μm) were equilibrated in aCSF at room temperature for at least 1 h before recording. The aCSF consisted of the following (in mM): 117 NaCl, 4.5 KCl, 2.5 CaCl_2_, 1.2 MgCl_2_, 1.2 NaH_2_PO_4_, 25 NaHCO_3_, and 11.4 dextrose bubbled with 95% O_2_/5% CO_2_, pH 7.4. During recordings, one slice was mounted on a submerged recording chamber and continuously perfused with oxygenated aCSF at 3–4 ml/min.

### Whole-cell patch-clamp recordings

The spinal lamina II was identified by a translucent band in the superficial dorsal horn on an upright fixed-stage IR-DIC microscope (BX51WI, Olympus, Tokyo, Japan). Spinal lamina I and outer lamina II were selected for whole-cell patch-clamp recordings, as previously described^7,46^. Glass pipette (resistance, 5–8 MΩ) were pulled and filled with an internal solution containing (in mM): 110 Cs^+^ gluconate, 5 TEA, 5 QX314, 0.5 CaCl_2_, 5 BAPTA, 10 HEPES, 5 MgATP, and 0.33 GTP-Tris, pH 7.3, 280 mOsm/L. The input resistance was monitored, and the recording was discarded if it changed more than 15%. All electrophysiological signals were acquired using an Axon setup (Molecular Devices/Axon Instruments, Union City, CA, USA). Signals were sampled by pCLAMP 9.2 via an amplifier (Axopatch 200B) and an AD-converter (Digidata 1322A), filtered at 2–5 kHz, digitized at 10 kHz, and stored for offline analysis. Miniature EPSCs (mEPSCs) were recorded at −70 mV in the presence of (−)bicuculline methiodide (10 μM), a GABA_A_ receptor antagonist with tetrodotoxin (1 μM).

### Small-interfering RNA

The 19-nucleotide siRNA duplex molecules used to target Munc13-1 and Fbxo45 were 5′-GCAAUGUGCUUCUCCAGUA-3′ and 5′-GGACAAUAAUCUACUACAU-3′, respectively, and the missense nucleotide sequence was 5′-UGAUAUUACCCUGAAUAUG-3′. The missense or siRNA construct was intrathecally administered using a polyethyleneimine (10 μL, Dharmacon, San Diego, CA)-based gene-delivery system into the dorsal subarachnoid space (L4–5) of animals through the implanted catheter (daily for 4 days).

### Drug application

TNF-α-neutralizing antibody (10, 30, and 100 ng, 10 μL, R&D, Minneapolis, USA) or TNF-α (1 pM, 10 μL, Sigma-Aldrich, Shanghai, China) was administered intrathecally by bolus injection. A vehicle solution of equal volume to that of the tested agents was dispensed to serve as a control.

### Data analysis

All data in this study were analyzed using Sigma Plot 10.0 (Systat Software, San Jose, CA) or Prism 6.0 (GraphPad, La Jolla, CA), and the results are expressed as the mean ± SEM. Paired two-tailed Student’s *t*-test was used to compare the means between groups. One-way or two-way ANOVAs were used to assess changes in values for serial measurements over time, and post hoc Tukey’s tests were used to compare the means of groups. Significance was set at *P* < 0.05.
